# Tuberculosis and HIV Co-Infection

**DOI:** 10.1371/journal.ppat.1002464

**Published:** 2012-02-16

**Authors:** Andrzej Pawlowski, Marianne Jansson, Markus Sköld, Martin E. Rottenberg, Gunilla Källenius

**Affiliations:** 1 Department of Clinical Science and Education, Karolinska Institutet, Stockholm, Sweden; 2 Department of Microbiology, Tumor and Cell Biology, Karolinska Institutet, Stockholm, Sweden; 3 Department of Laboratory Medicine, Lund University, Lund, Sweden; University of Alberta, Canada

## Abstract

Tuberculosis (TB) and HIV co-infections place an immense burden on health care systems and pose particular diagnostic and therapeutic challenges. Infection with HIV is the most powerful known risk factor predisposing for *Mycobacterium tuberculosis* infection and progression to active disease, which increases the risk of latent TB reactivation 20-fold. TB is also the most common cause of AIDS-related death. Thus, *M. tuberculosis* and HIV act in synergy, accelerating the decline of immunological functions and leading to subsequent death if untreated. The mechanisms behind the breakdown of the immune defense of the co-infected individual are not well known. The aim of this review is to highlight immunological events that may accelerate the development of one of the two diseases in the presence of the co-infecting organism. We also review possible animal models for studies of the interaction of the two pathogens, and describe gaps in knowledge and needs for future studies to develop preventive measures against the two diseases.

## Introduction

Tuberculosis (TB) and human immunodeficiency virus/acquired immune deficiency syndrome (HIV/AIDS) constitute the main burden of infectious disease in resource-limited countries. Estimates by the World Health Organization (WHO) indicate that there are more than 9 million new active cases of TB and close to 2 million deaths per year [1], and that 2.6 million new cases of HIV infection and 1.8 million AIDS-related deaths occur per year [2]. *Mycobacterium tuberculosis*–HIV co-infections pose particular diagnostic and therapeutic challenges and exert immense pressure on health care systems in African and Asian countries with large populations of co-infected individuals

In the individual host the two pathogens, *M. tuberculosis* and HIV, potentiate one another, accelerating the deterioration of immunological functions and resulting in premature death if untreated. Some 14 million individuals worldwide are estimated to be dually infected [3]. TB is the largest single cause of death in the setting of AIDS [4], accounting for about 26% of AIDS-related deaths [3], 99% of which occur in developing countries [5].

Both TB and HIV have profound effects on the immune system, as they are capable of disarming the host's immune responses through mechanisms that are not fully understood. HIV co-infection is the most powerful known risk factor for progression of *M. tuberculosis* infection to active disease, increasing the risk of latent TB reactivation 20-fold [3], [6]. Likewise, TB has been reported to exacerbate HIV infection [7], [8]. Various lines of evidence indicate that inborn errors of immunity, as well as genetic polymorphisms, have an impact on susceptibility to TB and HIV [9].

## Aspects of Immune Response to *M. tuberculosis* Infection

*M. tuberculosis* infects the host mainly through inhalation of aerosolized bacilli; alveolar macrophages are the primary target cells for this intracellular pathogen. Detection of *M. tuberculosis* by innate cells recognizing pathogen-associated molecular patterns, via toll-like receptors (TLRs) and nucleotide-binding oligomerization domain receptors, initiates a local inflammatory response and results in increased numbers of macrophages and dendritic cells (DCs) in infected lung tissue and draining pulmonary lymph nodes. Following activation by cytokines and innate receptor agonists, infected macrophages elicit direct bactericidal effector functions, such as reactive oxygen or nitrogen intermediates [10], [11], or expression of small GTPases that can regulate endosomal trafficking [12]. DCs can phagocytose the bacteria in lung tissue, migrate to draining lymph nodes, and initiate the adaptive immune response by priming naïve T lymphocytes [13].

Cell-mediated immunity is essential for control of *M. tuberculosis* infection; activation of both CD4^+^ and CD8^+^ T cells is seen in active TB in humans, as well as in mice after experimental infection [14]. CD4^+^ T lymphocytes of T helper cell type 1(Th1) are thought to be most critical [15]. Also, there is experimental evidence that CD8^+^ T cells [16], [17], as well as unconventional T cells such as CD1-restricted cells recognizing mycobacterial lipids [18], contribute to optimal control of the disease. T cells recruited to the infected lung are thought to control infection by producing interferon gamma (IFN-γ) in response to mycobacterial antigens presented by macrophages [19], [20]. In turn, IFN-γ activates macrophages to kill the intracellular bacteria through reactive nitrogen and oxygen intermediates [21], and by inducing phagolysosome formation [13]. However, these mechanisms might even be present in susceptible hosts, in which the infection progresses to disease. The full knowledge of the constituents of an effective protective immune response to TB is still incomplete.

In the *M. tuberculosis*–infected host there is also a robust humoral response, with a wide spectrum of antibodies (Abs) of different specificities and isotypes; although secondary to the cellular immune responses in terms of protection, B cells as well as certain Ab responses have been shown to be capable of playing an important role in protective immunity to TB [22].

## Aspects of Immune Response to HIV Infection

HIV-1, which most commonly infects via the genital mucosa, persists as a chronic infection even though the virus elicits strong innate and adaptive, including cellular and humoral, immunity. Explanations for this may be linked to virus genomic integration and subsequent cellular latency, as well as an extreme genetic variability, which translates into constant immune escape. HIV-specific CD8^+^ lymphocytes play a key role in the initial reduction of viremia during acute infection, but become increasingly dysfunctional and exhausted under conditions of chronic antigen persistence [23], [24]. Virus-neutralizing Abs are also elicited but are frequently accompanied by immune escape, and even if some individuals develop cross-neutralizing Abs, it is debatable whether Abs play a role in the control of the virus [25].

The hallmark of HIV infection is the depletion of CD4^+^ T cells. Interestingly, during the primary HIV infection, the cells that are preferentially depleted are the effector memory CD4^+^ T cells in the gut mucosa [26]. These immunopathogenic features, together with the systemic and chronic state of immune activation, including accelerated T cell turnover, are thought to contribute to progression of HIV disease [27]. Thus, constant antigenic stimulation is characterized by a dysfunctional T cell population displaying loss of functional potential, i.e., cytokine production and cytotoxic activity, and proliferative ability in response to antigen stimulation. In addition, the loss of immune balance between Th17 and regulatory T cells (Treg) during HIV disease progression has recently been implicated in permeabilization of gut integrity and the pathogenesis of HIV [28].

Microbial translocation caused by gut permeability has also been suggested to contribute to systemic immune activation observed during chronic HIV infection [29]. Hyper-responsiveness of plasmacytoid DCs during the primary infection, which results in excessive type-1 IFN production and the following chronic activation of these cells, may additionally contribute to systemic immune activation and HIV-1 disease progression [30].

CD8^+^ T cells have been implicated in the control of chronic HIV replication as suggested by studies on simian immunodeficiency virus (SIV) viremia in non-human primates after in vivo CD8^+^ T cell depletion [31]. In addition, there are rare individuals who control HIV-1 replication to undetectable levels, i.e., elite controllers. This phenotype is strongly associated with some MHC class I alleles [32] and with the presence of HIV-specific CD8^+^ T cells showing superior cytotoxic capacity to kill HIV-infected targets [33], [34].

Programmed-Death 1 (PD-1) and T cell immunoglobulin and mucin domain 3 (Tim-3) are two examples of markers of T cell exhaustion in HIV-1^+^ patients caused by constant antigenic stimulation [35], [36]. Both molecules are involved in the down-regulation of host immune responses and play a role in maintaining T cell tolerance. A recent finding is that Tim-3 was up-regulated on virus-specific CD8^+^ T cells in patients with chronic progressive HIV infection [36]; another recent publication reports that Tim-3 was up-regulated on antigen-specific CD8^+^ T cells in patients with active TB [37], indicating that similar inhibitory receptor/ligand interactions play a role in modulating host immunity to both HIV and *M. tuberculosis* infections in humans.

## TB Reactivation by HIV

It is generally thought that one-third of the world's population is latently infected with *M. tuberculosis* [38], although the data supporting this notion may be questioned. Also, the rate of progression from infection to disease varies greatly. Approximately 10% of *M. tuberculosis*–infected individuals are thought to develop overt clinical disease [6] and about half of them develop disease more than two years after infection; these cases are commonly named “reactivation” or post-primary TB [39]. Thus, the lifetime risk of developing active TB in immunocompetent adults is estimated to be 5%–10% during their lifetime, but in HIV-positive individuals this risk is increased to 5%–15% annually [40].

The depletion of CD4^+^ T cells, which is a main feature of AIDS, is certainly an important contributor to the increased risk of reactivation of latent TB and susceptibility to new *M. tuberculosis* infection. There is also some evidence that CD8^+^ T cells play a role in the control of latent TB [41]–[44]. Other mechanisms reported to facilitate *M. tuberculosis* infection and disease in individuals with HIV are up-regulation of *M. tuberculosis* entry receptors on macrophages [45], HIV manipulation of macrophage bactericidal pathways [46], deregulated chemotaxis [47], and a tipped Th1/Th2 balance [48]. It has also been shown that HIV impairs tumor necrosis factor (TNF)-mediated macrophage apoptotic response to *M. tuberculosis* and thus facilitates bacterial survival [49].

In the latent phase of TB, the bacteria are not completely eradicated despite a seemingly robust Th1 immune response. A failure or an alteration of the quality or levels of the protective adaptive immune responses or of the cross-talk with innate immune responses leads to reactivation of infection. Several immune mechanisms, such as increased levels of FoxP3^+^ Treg cells [50], increased production of IL-27 [51], TGF-β [52], [53], PGE-2 [54], SOCS1, or the decoy receptor D6 [55], or diminished levels of IFN-γ, TNF, and polyfunctional specific T cells, are believed to play a role in such reactivation. Many of these factors, such as SOCS1 or IL-27, down-regulate the IFN-γ/IL-12 axis, thereby impairing bacterial control, while others, such as the D6 decoy receptor, are mainly anti-inflammatory, but may indirectly inhibit efficient bacterial clearance. Some of these mechanisms may also underlie HIV-infected patients' increased susceptibility to active TB.

Granulomas are organized cellular structures that constitute TB's pathologic hallmark. Mycobacteria are contained within the granuloma, which, by localizing infection and thus potentially preventing spread of the disease between hosts, probably contributes to protection. CD4^+^ T cells and TNF are important in maintaining granuloma organization. Granuloma formation may fail in individuals with a compromised immune system, and there are several hypotheses about how HIV exacerbates TB pathology through the manipulation of granulomas [56]. Specifically, TB patients with AIDS present a dominant granulocytic infiltrate and necrosis without the typical caseous necrosis seen in non-HIV-infected TB granulomas. This has been associated with the killing of CD4^+^ cells in the granuloma, probably resulting in a direct disruption of granuloma structure and abolition of the containment of infection. Cavitary lesions are seldom encountered in patients with a CD4 T-lymphocyte count <200/mm^3^ [57]. As a result, while in the majority of adult patients TB is confined preferentially to the lungs, in HIV-infected patients TB can be a systemic disease involving multiple organs that lack well-defined granulomas and instead develop more diffuse lesions [58]. All forms of extrapulmonary TB have been described in patients with HIV.

In macaques, SIV induces distortions in pro-inflammatory and anti-inflammatory T cell responses within the granuloma that may have significant effects on reactivation of latent TB. Reduction of T cell numbers also occurred within lung granulomas of monkeys co-infected with SIV compared with monkeys exclusively infected with TB [59]. It is important to note that besides the known increased risk of disseminated disease in adults with HIV, there is a growing recognition from prevalence surveys of subclinically active TB infection in co-infected individuals [60].

## Exacerbation of HIV Infection by *M. tuberculosis* Infection

The incidence and mortality rates for new AIDS-defining opportunistic infections have been shown to be higher if individuals with HIV are co-infected with TB [7], [61]. Despite these epidemiological data supporting the notion that *M. tuberculosis* infection has a negative impact on the immune response to HIV and on progression to AIDS, research on possible mechanisms is scarce. The function of many immune cells, including macrophages and DCs, is modulated by both HIV and *M. tuberculosis*. Increased replication of the virus was demonstrated locally, at sites of *M. tuberculosis* infection in the lung [62], and within activated cells, including lymphocytes and CD14^+^ macrophages, of the pleural space [63] of co-infected patients. *M. tuberculosis* has been reported to up-regulate HIV-1 replication in chronically or acutely infected T cells or macrophages [64], [65], as well as ex vivo in alveolar macrophages and lymphocytes from patients with HIV [66], [67]. These in vitro/ex vivo findings are also reflected in vivo where elevated plasma viral loads have been detected in HIV-infected individuals with concomitant active TB disease [68].

The primary target for *M. tuberculosis*, the alveolar macrophage, can also be infected with HIV [69]–[71]. Mycobacteria exacerbate HIV replication in macrophages and lung cells obtained by bronchoalveolar lavage from co-infected individuals [62], [65], [72]. Also, in vitro studies have demonstrated that *M. tuberculosis* infection can up-regulate both HIV infection and replication within monocyte-derived macrophages (MDMs), increase the efficiency of virus transmission from infected MDMs to T cells, and favor replication of X4 HIV variants by up-regulation of CXCR4 [73]. Furthermore, monocytes from HIV^+^ patients display an impaired response to TLR ligands [74], and viral proteins can interfere with both MDM and DC maturation and function in vitro, including their ability to phagocytose mycobacteria and kill intracellular bacteria [75]–[78]. SOCS1, which is stimulated by infection with *M. tuberculosis* [45], has been shown to facilitate the late replication pathways of HIV infection [79] and mediate viral evasion of type I IFN anti-viral signalling [80].

While TNF production in response to *M. tuberculosis* infection is required for control of bacterial growth, TNF is known to activate HIV replication in macrophages [81], indicating that the host immune response initiated against one pathogen may promote the replication of another. Thus, both HIV and *M. tuberculosis* stimulate TNF release from infected cells, and TNF hampers bacterial growth while enhancing HIV replication.

*M. tuberculosis* survives in DCs and actively down-regulates their pro-inflammatory activity and antigen-presenting function, with concurrent induction of anti-inflammatory cytokines [82]. Similarly, HIV can infect and also manipulate DCs and the ensuing T cell functions [83]. In HIV infection, not only is DC-mediated activation of T cells impaired, but the migration of infected DCs can also contribute to pathogen dissemination.

The DC-expressed C-type lectin receptor DC-SIGN (DC-specific intercellular-adhesion-molecule-3-grabbing nonintegrin) has been suggested to facilitate transmission and immune escape of both *M. tuberculosis* and HIV [84]. HIV attaches to DC-SIGN through interaction with the viral envelope glycoprotein gp120, and this interaction is thought to contribute to efficient spread and transmission of the virus to CD4^+^ T cells in *trans* [85], [86]. *M. tuberculosis* has been reported to target DC-SIGN by a mechanism that is distinct from that of HIV, leading to inhibition of pro-inflammatory IL-12 and TNF production and induction of IL-10 by DCs [87] and, hence, down-regulation of protective immune responses.

It has been suggested that TB patients have a microenvironment that facilitates HIV infection by *i*) increasing the expression of co-receptors CXCR4 and CCR5 regulated by *M. tuberculosis* products; *ii*) increasing pro-inflammatory cytokines, especially TNF; and *iii*) down-regulation of CCL5 [45]. It has also been shown that *M. tuberculosis* and its cell wall component, lipoarabinomannan (LAM) [88], may activate replication of HIV in provirus-carrying cells by inducing TNF and IL-6 production through the NF-κB pathway, which in turn triggers transcriptional activation of the long terminal repeat (LTR) promoter and supports replication of HIV [5].

## Immune Reconstitution Inflammatory Syndrome

A particularly intriguing phenomenon is immune reconstitution inflammatory syndrome (IRIS). IRIS may develop in *M. tuberculosis* and HIV co-infected patients who undergo anti-TB treatment and antiretroviral therapy (ART) [89], [90]. The definition of IRIS in TB/HIV co-infected patients is still debated [91]. The patients present with an exacerbation of symptoms and radiological manifestations of TB, and recognized predictors of IRIS include low CD4^+^ T lymphocyte counts and high plasma viral load prior to initiation of ART, and an increase in CD4^+^ counts after highly active antiretroviral therapy (HAART) onset [92]. Possible mechanisms responsible for IRIS may be a sustained Th1-response against mycobacterial antigens, which is followed by dysregulation of cytokine secretion and T cell migration to the inflammatory site [93]. Recently, it was shown that patients who developed IRIS had higher pre-ART levels of TNF and increasing levels of inflammation biomarkers [94]. Moreover, it has been demonstrated that TB/HIV co-infected patients who experienced IRIS had significantly lower levels of Abs to the phenolic glycolipid (PGL-TB1) antigen, specific for *M. tuberculosis*, compared to patients who did not develop TB-IRIS [95].

## Animal Models to Study *M. tuberculosis* and HIV Co-Infection

One of the most important challenges in studies of co-infection is to identify appropriate animal models, since HIV does not cause disease in rodents or even non-human primates. Thus, while mice are ideal models to study immune response to infection and vaccination due to the large diversity of tools and knowledge about their immune system, conventional mice are not susceptible to HIV infection owing to the restricted specificity of the virus for the human cell. To circumvent this limitation, two complementary mouse models were recently generated. Using these models, the most relevant features of *M. tuberculosis* and HIV infections can be reproduced in mice (e.g., typical TB granuloma formation; virus replication in splenic lymphocytes, peritoneal macrophages, and brain; immune exhaustion and/or chronic immune activation; and susceptibility to systemic, vaginal, and rectal HIV infection). In one, the “humanized mouse”, human hematopoietic progenitor cells (CD34^+^) from human cord blood are used to reconstitute the immune system of immunodeficient mice [96]. A variation of this model incorporates the engraftment of a fetal human thymus fragment, which gains activity as a functional human thymus, allowing a more proper positive and negative T cell selection than the original model [97]. Features such as CD4^+^ cell depletion, prolonged viremia, and co-receptor-mediated tropism were observed during HIV infection of humanized mice [97]–[101]. These mice show transplanted human cells in mucosal surfaces and could thereby be infected by the intravaginal and intrarectal routes [102]. The model has been used to evaluate new approaches to the prevention or treatment of HIV infection, including human-neutralizing Abs [102], prophylactic usage of antiretrovirals [98], and T cell–specific siRNA delivery [103]. Inoculation of humanized mice with mycobacteria enhances the CD4/CD8 cell ratio, the differentiation of CD4^+^ cells into memory/effector types, and the translocation of IFN-γ-secreting T cells into the lung. Of particular importance is that lungs and livers from infected mice show typical features of mycobacterial granuloma (F. Heuts, D. Gavier Widén, B. Carow, J. Juarez, H. Wigzell, et al., unpublished data). On the negative side, the adaptive immune responses, especially the specific IgG levels in response to immunization or infection, are low.

The other mouse model used to study *M. tuberculosis*/HIV interaction, the “EcoHIV” model, makes use of a modified HIV-1 strain, in which the coding region of gp120 has been replaced by that of gp80 from ecotropic murine leukemia virus, that is able to infect the immune cells of conventional mice [104]. The resulting chimeric virus construct, EcoHIV, productively infects immunocompetent mice. Replicating virus is detected in splenic lymphocytes, peritoneal macrophages, and the brain of mice. The chimeric virus also elicits an immune response directed against viral proteins, and stimulates mouse genes similar to those stimulated by HIV in humans. This murine model of HIV infection has proven useful in vaccine challenge studies and for preclinical evaluation of antiretroviral drugs [105]–[107].

HIV transgenic mice incorporating the entire viral genome have also been used to study the effect of *M. tuberculosis* infection on the induction of HIV gene expression [108]. In this model, viral gene expression was activated by *M. tuberculosis* and suppressed after anti-mycobacterial chemotherapy [108].

Although non-human primates are resistant to infection by HIV, they can be infected by SIV, a retrovirus causing immunodeficiency similar to AIDS in Asian macaques. Thus, SIV infection in macaques has been used as a model for AIDS. Macaques also develop TB that is very similar to TB in humans, and can develop cavitary lung disease and necrotic lesions. They also can maintain TB latency for years, and only a small proportion of latently infected macaques develop reactivation [56]. Macaques infected with SIV can develop persistent *Mycobacterium bovis* bacillus Calmette Guerin (BCG) [109] and *M. tuberculosis* co-infection [59], [110]. Co-infection with SIV and BCG accelerated progression to AIDS [111] and coincided with a severe depletion of CD4^+^ T cells, loss of BCG-specific T cell responses, and reactivation of the clinically latent BCG infection into a TB-like disease [112]. *M. tuberculosis* reactivation in SIV-infected macaques is associated with early peripheral T cell depletion and not virus load [59].

## Summary and Perspectives

TB/HIV co-infection represents a novel pathogenic scenario at the global level. It constitutes a serious diagnostic and therapeutic challenge and, particularly in poor countries, weighs heavily on already strained health care budgets. It has recently been realized that the epidemiology, clinical manifestations, and management of both HIV and *M. tuberculosis* infections are different and far more complex in co-infected compared to mono-infected patients. However, our knowledge about the mechanisms of interaction of the two pathogens still has many gaps that need to be filled in order to develop preventive measures against the two diseases (Box 1).

**Box 1. Scientific and Technological Objectives for Integrating Knowledge in the Field of Co-Infections with HIV and *M. tuberculosis***

- Development of standardized in vitro and in vivo models for studies of co-infections
- Interactions and receptor signalling in dendritic cells
- HIV/*M. tuberculosis*-specific T/B cell responses
- Role of memory T cells in the maintenance of latent infection and of regulatory T cells in disease outbreak
- Effector mechanisms of T cells involved in protection against TB
- The role of antimicrobial peptides in cytolytic T cells
- Regulation of T cell differentiation during co-infection
- Immunological synapse; interactions of T and antigen-presenting cells
- Mathematical modelling and simulation of T, B, and NK cell repertoires
- Mechanisms of HIV–TB interactions in IRIS
- The role of individual HIV/*M. tuberculosis* antigens/molecules in immunopathology
- Effect on immune response in infected individuals after vaccination with TB and/or HIV vaccine candidates

Ultimately, the most cost-effective way of combating the two diseases would be vaccination. The present TB vaccine, BCG, does not effectively prevent the most prevalent form of the disease, pulmonary TB in adults. Similarly, no effective, preventive HIV vaccine can be discerned on the horizon, although many vaccine candidates are being evaluated in clinical trials. One approach would be to construct a combined TB/HIV vaccine [113], such as a recombinant BCG vaccine as a vehicle for combinations of mycobacterial and HIV antigens.

The design of candidate vaccines is, however, a particularly difficult task since laboratory correlates of protection have not been defined for *M. tuberculosis* and HIV infections. In addition, vaccine-induced immune responses need to be tipped towards protection, avoiding those that may result in immunopathology; this requires meticulous study of appropriate adjuvants, antigens, and vaccination regimens for the novel vaccines. Here the immunomodulatory role of individual antigens of the two pathogens needs further elucidation. For example, the major HIV antigen gp120 [114] and mycobacterial compounds such as glycolipids of the cell wall, particularly LAMs, PIMs, and phenolic glycolipids [115], play a crucial role in modulating immune responses. It is also increasingly apparent that these compounds may differ in biologic activity depending on strain lineages of the two pathogens [53], [115], [116].

Since both pathogens enter the host through mucosal surfaces, a combination vaccine given at mucosal sites would probably be optimal [117]–[119]. However, for this, further research in the biology of concurrent *M. tuberculosis* and HIV infections is urgently needed, using in vitro systems, animal models, and clinical studies, as well as vaccine trials.

Thus, an integrated approach to the two diseases should lead to novel concepts and correlates of protection and to the identification of antigen targets useful for new therapies to overcome the rapidly increasing drug resistance of both diseases, as well as for vaccination.
